# Dysregulation of Neuronal Gαo Signaling by Graphene Oxide in Nematode *Caenorhabditis elegans*

**DOI:** 10.1038/s41598-019-42603-1

**Published:** 2019-04-15

**Authors:** Peidang Liu, Huimin Shao, Xuecheng Ding, Ruilong Yang, Qi Rui, Dayong Wang

**Affiliations:** 1 Medical School, Southeast University, Nanjing, 210009 China; 2 College of Life Sciences, Nanjing Agricultural University, Nanjing, 210095 China

## Abstract

Exposure to graphene oxide (GO) induced some dysregulated microRNAs (miRNAs), such as the increase in *mir-247*, in nematode *Caenorhabditis elegans*. We here further identified *goa-1* encoding a Gαo and *pkc-1* encoding a serine/threonine protein kinase as the targets of neuronal *mir-247* in the regulation of GO toxicity. GO exposure increased the expressions of both GOA-1 and PKC-1. Mutation of *goa-1* or *pkc-1* induced a susceptibility to GO toxicity, and suppressed the resistance of *mir-247* mutant to GO toxicity. GOA-1 and PKC-1 could also act in the neurons to regulate the GO toxicity, and neuronal overexpression of *mir-247* could not affect the resistance of nematodes overexpressing neuronal *goa-1* or *pkc-1* lacking 3′-UTR to GO toxicity. In the neurons, GOA-1 acted upstream of diacylglycerol kinase/DGK-1 and PKC-1 to regulate the GO toxicity. Moreover, DGK-1 and GOA-1 functioned synergistically in the regulation of GO toxicity. Our results highlight the crucial role of neuronal Gαo signaling in response to GO in nematodes.

## Introduction

Short noncoding microRNAs (miRNAs) exist in many organisms, including human. miRNAs usually regulate various biological processes by suppressing expression and function of certain targeted genes post-transcriptionally^1,2^. The founding members of miRNA family, such as *lin-4* and *let-7*, were firstly identified in *Caenorhabditis elegans via* forward genetic screen^3^. Due to sensitivity to environmental toxicants or stresses^4–6^, *C*. *elegans* has been widely used in toxicity assessment and toxicological study of various engineered nanomaterials (ENMs)^7–9^. Moreover, certain amount of miRNAs in response to ENMs, such as carbon-based ENMs, have been identified in nematodes^10,11^.

Graphene oxide (GO), a member of graphenic nanomaterials, can be potentially applied in at least drug delivery, biosensors, bioimaging, cancer therapy, catalytic, and environmental decontamination due to its unique physical and chemical properties^12,13^. With the increase in these potential applications, availability of GO to both human and environmental organisms has received the great attention recently. Some *in vitro* and *in vivo* studies have demonstrated the cytotoxicity, the pulmonary toxicity, and reproductive toxicity of GO^14–16^. In nematodes, exposure to GO resulted in damage on the functions of both primary targeted organs (such as intestine) and secondary targeted organs (such as neurons and reproductive organs)^6^.

Among the dysregulated miRNAs in GO exposed nematodes, *mir-247* is the homologous of human *miR-134* and *miR-708*^17^, and the expression of *mir-247* was up-regulated by GO exposure^18^. Mutation of *mir-247* induced a resistance to GO toxicity^18^. *mir-247* could act in the neurons to regulate the GO toxicity, and neuronal overexpression of *mir-247* resulted in a susceptibility to GO toxicity^18^. However, molecular mechanism for the role of neuronal *mir-247* in the regulation of GO toxicity is still unclear. Therefore, we here further employed *C*. *elegans* to investigate the underlying mechanism for the function of neuronal *mir-247* in regulating GO toxicity. We here identified GOA-1, an ortholog of heterotrimeric G protein α subunit Gαo, as a downstream target for neuronal *mir-247* in regulating GO toxicity. Moreover, in the neurons, a signaling cascade of GOA-1-DGK-1/PKC-1 was raised to explain the underlying mechanism for induction of GO toxicity. Our study provides an important molecular basis for neuronal Gαo signaling in response to GO in organisms.

## Results

### GOA-1, PKC-1, and CEH-18 might act as the possible targets for neuronal *mir-247* in the regulation of GO toxicity

We employed TargetScan software (http://www.targetscan.org/worm_52/) with preferentially conserved targeting (PCT) between 0 and 1 and miRBase (http://www.mirbase.org) with a score threshold of −0.1 to predict potential targets for *mir-247* in the regulation of GO toxicity by searching for the presence of conserved sites that match seed region of *mir-247*^19,20^. Among the searched genes possibly acting as the targets for *mir-247*^18^, *goa-1*, *gpb-2*, *tcer-1*, *eat-16*, *rps-8*, *lin-41*, *cwn-1*, *vhp-1*, *rab-39*, *ceh-18*, *acy-1*, *daf-19*, *pkc-1*, *lrk-1*, *cnb-1*, *dve-1*, *acs-22*, *egl-15*, *kin-1*, *mdt-15*, *apr-1*, and *dkf-2* are associated with the regulation of stress response in nematodes^21–40^. The anticipated phenotype of nematodes with mutation of targeted gene is susceptible to GO toxicity. However, mutation of *cwn-1* or *apr-1* was resistant to GO toxicity in nematodes^35,40^.

Our previous study has suggested that *mir-247* acts in the neurons to regulate GO toxicity^18^. Among the other 20 possible targeted genes of *mir-247*, *goa-1*, *gpb-2*, *tcer-1*, *eat-16*, *lin-41*, *vhp-1*, *rab-39*, *ceh-18*, *acy-1*, *daf-19*, *pkc-1*, *lrk-1*, *cnb-1*, *dve-1*, *acs-22*, *egl-15*, *kin-1*, *mdt-15*, and *dkf-2* can be expressed in the neurons (http://www.wormbase.org/species/c_elegans/gene). The working concentration (10 mg/L) was selected as described previously^18^. Prolonged exposure to GO (10 mg/L) did not affect expressions of *gpb-2*, *tcer-1*, *lin-41*, *vhp-1*, *rab-39*, *acy-1*, *daf-19*, *lrk-1*, *cnb-1*, *acs-22*, *egl-15*, *kin-1*, *mdt-15*, and *dkf-2* (Fig. S1a). In contrast, GO (10 mg/L) decreased expressions of *goa-1*, *eat-16*, *ceh-18*, and *pkc-1* and increased the transcriptional expression of *dev-1* (Fig. S1a). Since GO exposure could increase the *mir-247* expression^18,41^, the anticipated expression tendency of targeted genes after GO exposure should be suppression in wild-type nematodes.

We next examined the expressions of *goa-1*, *eat-16*, *ceh-18*, and *pkc-1* in GO exposed nematodes overexpressing the neuronal *mir-247*. Neuronal overexpression of *mir-247* could not obviously affect the transcriptional expression of *eat-16* after GO (10 mg/L) exposure (Fig. S1b). In contrast, neuronal overexpression of *mir-247* could significantly suppress the transcriptional expressions of *goa-1*, *ceh-18*, and *pkc-1* after GO (10 mg/L) exposure (Fig. S1b). Therefore, *goa-1*, *ceh-18*, and *pkc-1* might be the targeted genes for neuronal *mir-247* in the regulation of GO toxicity. In *C*. *elegans*, *goa-1* encodes an ortholog of the heterotrimeric G protein α subunit, *ceh-18* encodes a POU-class homeodomain transcription factor, and *pkc-1* encodes a serine/threonine protein kinase.

### Mutation of *goa-1* or *pkc-1* induced a susceptibility to GO toxicity

Using the *goa-1(sa734)*, *ceh-18(ok1082)*, and *pkc-1(ok563)* mutants, we next investigated the possible function of GOA-1, CEH-18, and PKC-1 in the regulation of GO toxicity. Under the normal conditions, mutation of *goa-1*, *ceh-18*, or *pkc-1* did not induce the intestinal ROS production and affect the locomotion behavior (Fig. S2). In nematodes, mutation of *ceh-18* did not influence the toxicity of GO (10 mg/L) (Fig. S2). In contrast, mutation of *goa-1* or *pkc-1* resulted in a susceptibility to GO toxicity (Fig. S2). Therefore, both GOA-1 and PKC-1 may negatively regulate the GO toxicity.

### Genetic interaction between *mir-247* and *goa-1* or *pkc-1* in the regulation of GO toxicity

**Figure 1:**
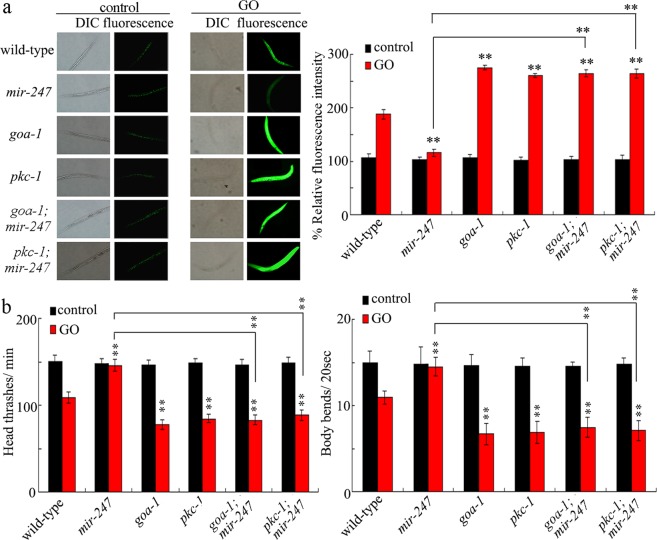
Genetic interaction between *mir-247* and *goa-1* or *pkc-1* in the regulation of GO toxicity. (**a**) Genetic interaction between *mir-247* and *goa-1* or *pkc-1* in the regulation of GO toxicity in inducing intestinal ROS production. (**b**) Genetic interaction between *mir-247* and *goa-1* or *pkc-1* in the regulation of GO toxicity in decreasing locomotion behavior. GO exposure concentration was 10 mg/L. Prolonged exposure was performed from L1-larvae to adult day-1. Bars represent means ± SD. ^**^P < 0.01 *vs* wild-type (if not specially indicated).

To confirm the molecular interaction between *mir-247* and *goa-1* in the regulation of GO toxicity, we compared the GO toxicity in *goa-1(RNAi);mir-247(n4505)* with that in *mir-247(n4505)* or *goa-1(RNAi)*. After GO exposure, the toxicity in *goa-1(RNAi);mir-247(n4505)* was similar to those in *goa-1(RNAi)* (Fig. 1). We also compared the GO toxicity in *pkc-1(RNAi);mir-247(n4505)* with that in *mir-247(n4505)* or *pkc-1(RNAi)*. Similarly, after GO exposure, the toxicity in *pkc-1(RNAi);mir-247(n4505)* was similar to those in *pkc-1(RNAi)* (Fig. 1). Therefore, the neuronal *mir-247* may suppress the function of both GOA-1 and PKC-1 in the regulation of GO toxicity.

### Neuronal overexpression of *mir-247* could not affect the resistance of nematodes overexpressing neuronal *goa-1* or *pkc-1* lacking 3′-UTR to GO toxicity

**Figure 2:**
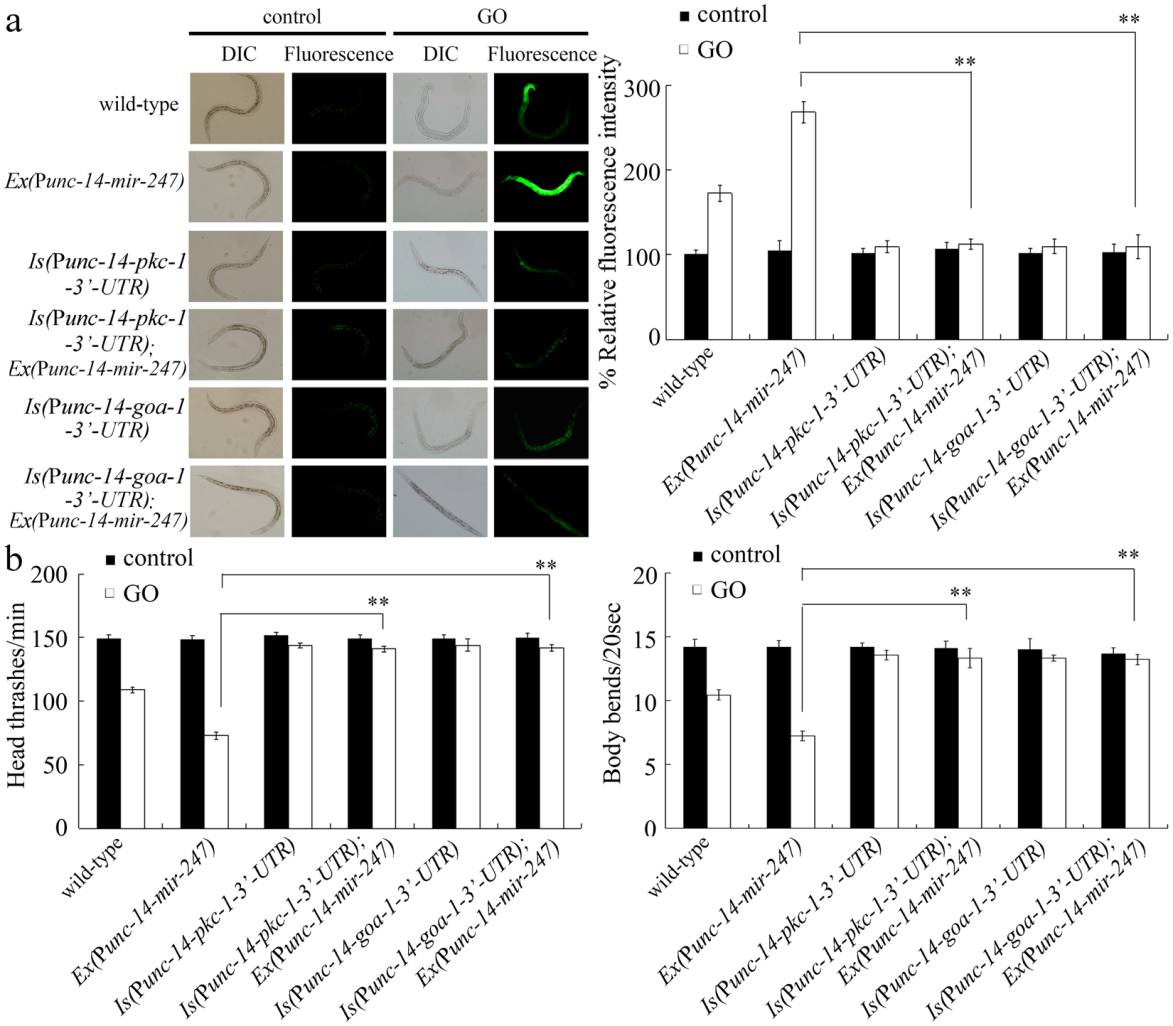
Effects of neuronal overexpression of *mir-247* on GO toxicity in nematodes overexpressing neuronal *goa-1* or *pkc-1* lacking 3′-UTR. (**a**) Effects of neuronal overexpression of *mir-247* on GO toxicity in inducing intestinal ROS production in nematodes overexpressing neuronal *goa-1* or *pkc-1* lacking 3′-UTR. (**b**) Effects of neuronal overexpression of *mir-247* on GO toxicity in decreasing locomotion behavior in nematodes overexpressing neuronal *goa-1* or *pkc-1* lacking 3′-UTR. GO exposure concentration was 10 mg/L. Prolonged exposure was performed from L1-larvae to adult day-1. Bars represent means ± SD. ^**^P < 0.01.

To further confirm the roles of GOA-1 and PKC-1 as the target of neuronal *mir-247*, we next investigated the genetic interaction between *mir-247* and *goa-1* or *pkc-1* in the neurons to regulate the GO toxicity. We introduced the *goa-1* or *pkc-1* lacking 3′-UTR driven by *unc-14* promoter into the nematodes overexpressing neuronal *mir-247*. After GO exposure, the transgenic strain *Is(*P*unc-14-goa-1-3*′*-UTR);Ex(*P*unc-14-mir-247)* exhibited the similar resistance to GO toxicity to that in the transgenic strain *Is(*P*unc-14-goa-1-3*′*-UTR)* (Fig. 2). Additionally, the transgenic strain *Is(*P*unc-14-pkc-1-3*′*-UTR);Ex(*P*unc-14-mir-247)* showed the similar resistance to GO toxicity to that in the transgenic strain *Is(*P*unc-14-pkc-1-3*′*-UTR)* (Fig. 2).

### Tissue-specific activity of *goa-1* in the regulation of GO toxicity

**Figure 3:**
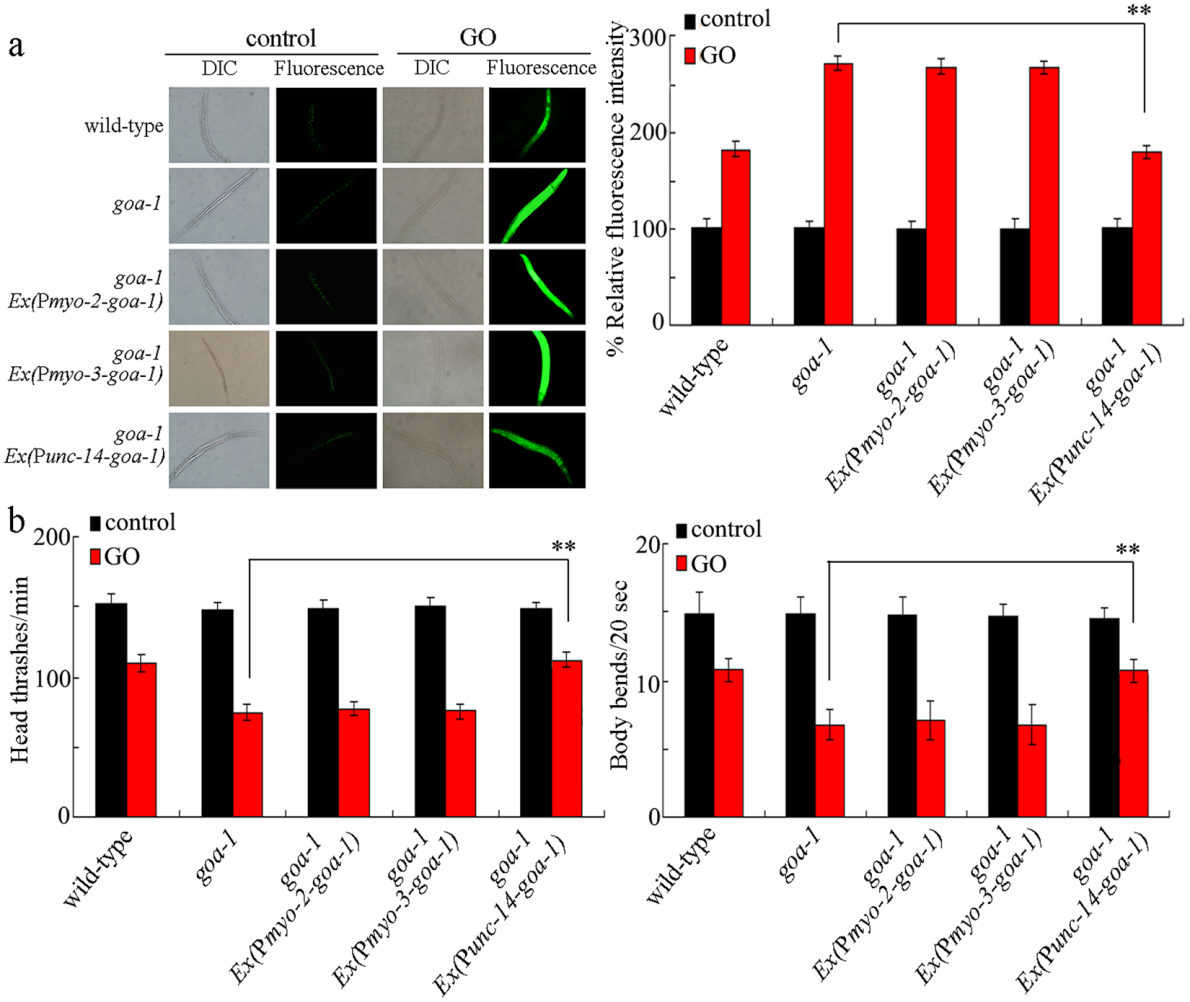
Tissue-specific activity of *goa-1* in the regulation of GO toxicity in nematodes. (**a**) Tissue-specific activity of *goa-1* in the regulation of GO toxicity in inducing intestinal ROS production. (**b**) Tissue-specific activity of *goa-1* in the regulation of GO toxicity in decreasing locomotion behavior. GO exposure concentration was 10 mg/L. Prolonged exposure was performed from L1-larvae to adult day-1. Bars represent means ± SD. ^**^P < 0.01 *vs goa-1*.

*goa-1* gene is expressed in the pharynx, the neurons, and the muscle^42,43^. *pkc-1* is only expressed in the neurons^44^. Using tissue-specific promoters, we investigated the tissue-specific activity of *goa-1* in the regulation of GO toxicity. Rescue assay by expression of *goa-1* in the pharynx or the muscle did not significantly influence the susceptibility of *goa-1(sa734)* mutant to GO toxicity (Fig. 3). Different from these, expression of *goa-1* in the neurons could significantly suppress the susceptibility of *goa-1(sa734)* mutant to GO toxicity (Fig. 3). These results suggest that both GOA-1 and PKC-1 may act in the neurons to regulate the GO toxicity.

### Identification of downstream targets for GOA-1 in the Gαo signaling pathway in the regulation of GO toxicity

**Figure 4:**
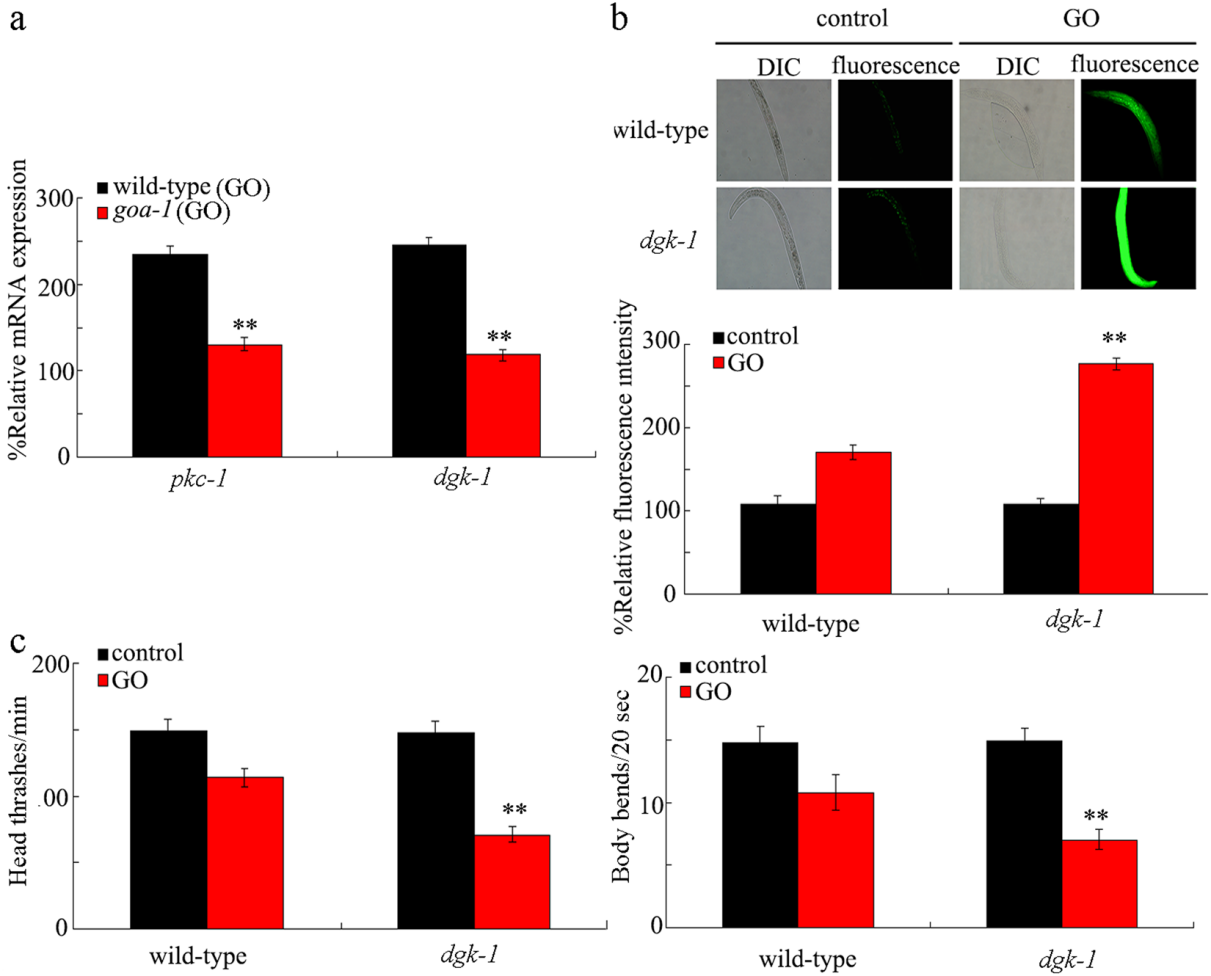
Effects of *dgk-1* mutation on GO toxicity in nematodes. (**a**) Effect of *goa-1* mutation on expressions of *pkc-1* and *dgk-1* in GO exposed nematodes. Bars represent means ± SD. ^**^P < 0.01 *vs* wild-type (GO). (**b**) Effect of *dgk-1* mutation on GO toxicity in inducing intestinal ROS production. Bars represent means ± SD. ^**^P < 0.01 *vs* wild-type. (**c**) Effect of *dgk-1* mutation on GO toxicity in decreasing locomotion behavior. GO exposure concentration was 10 mg/L. Prolonged exposure was performed from L1-larvae to adult day-1. Bars represent means ± SD. ^**^P < 0.01 *vs* wild-type.

In the Gαo signaling pathway, DGK-1 is a downstream target for GOA-1, and *dgk-1* encodes an ortholog of mammalian diacylglycerol kinase theta (DGKQ)^45^. In GO (10 mg/L) exposed *goa-1* mutant, we detected the significant decrease in expressions of both *pkc-1* and *dgk-1* compared with those in GO (10 mg/L) exposed wild-type nematodes (Fig. 4a), which implies that both PKC-1 and DGK-1 may act as important downstream targets for GOA-1 during the control of GO toxicity.

Using induction of intestinal ROS production and locomotion behavior as the toxicity assessment endpoints, we found that the *dgk-1(sy428)* mutant was susceptible to GO toxicity (Fig. 4b,c), suggesting that GOA-1 may positively regulate GO toxicity by affecting functions of PKC-1 and DGK-1.

### Genetic interaction between GOA-1 and PKC-1 or DGK-1 in the regulation of GO toxicity

**Figure 5:**
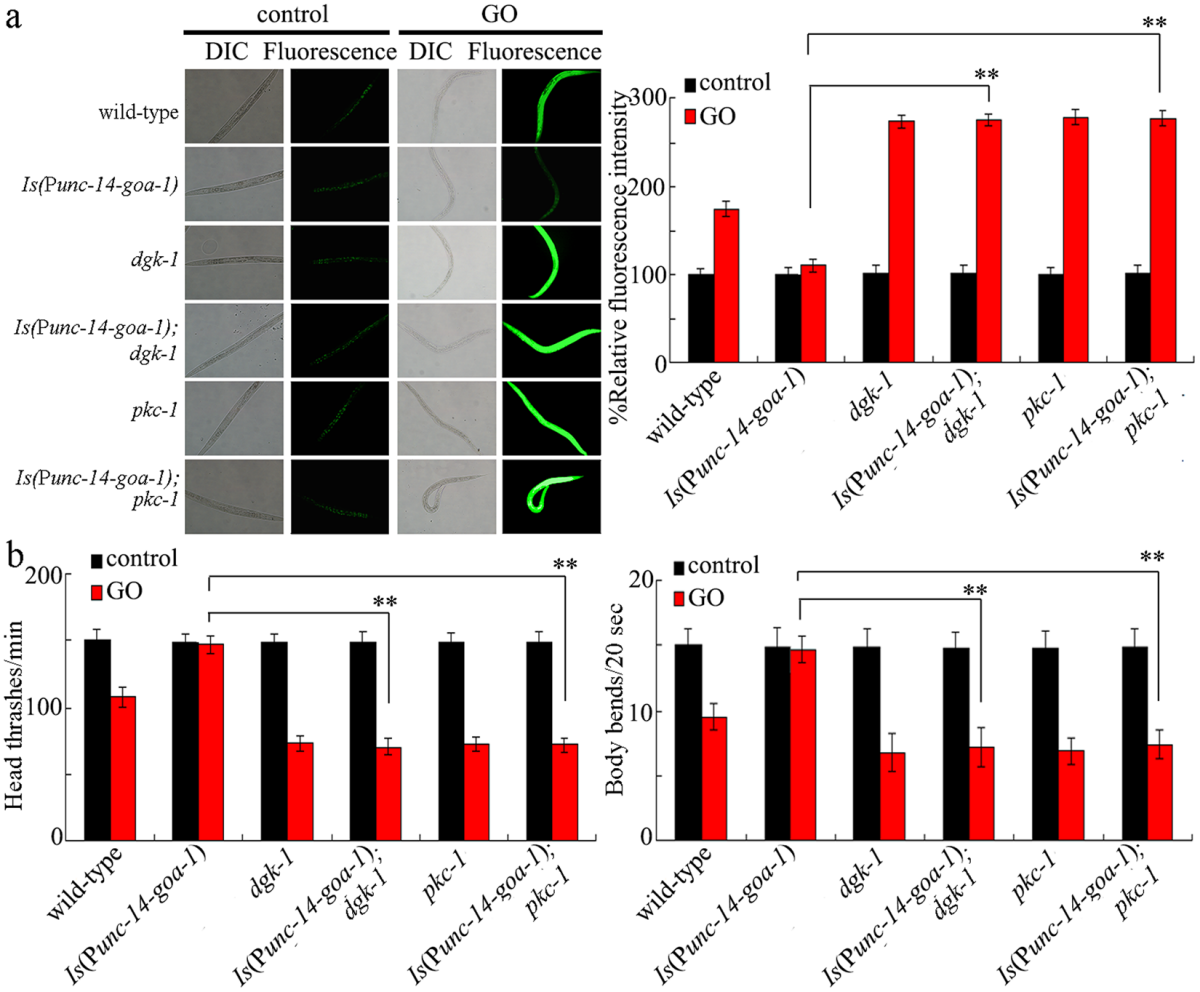
Genetic interaction between GOA-1 and PKC-1 or DGK-1 in the regulation of GO toxicity. (**a**) Genetic interaction between GOA-1 and PKC-1 or DGK-1 in the regulation of GO toxicity in inducing intestinal ROS production. (**b**) Genetic interaction of GOA-1 and PKC-1 or DGK-1 in the regulation of GO toxicity in decreasing locomotion behavior. GO exposure concentration was 10 mg/L. Prolonged exposure was performed from L1-larvae to adult day-1. Bars represent means ± SD. ^**^P < 0.01 *vs Is* (P*unc-14-goa-1*).

To determine the genetic interaction between *goa-1* and *dgk-1* or *pkc-1* in the regulation of GO toxicity, we examined the effects of mutation of *dgk-1* or *pkc-1* on GO toxicity in transgenic strain overexpressing the neuronal *goa-1*. The nematodes overexpressing neuronal *goa-1* exhibited the resistance to GO toxicity (Fig. 5). In contrast, after the GO exposure, *dgk-1* or *pkc-1* mutation suppressed the resistance of nematodes overexpressing neuronal *goa-1* to GO toxicity (Fig. 5). Therefore, neuronal GOA-1 may act upstream of both DGK-1 and PKC-1 to regulate the GO toxicity.

### Genetic interaction between PKC-1 and DGK-1 in the regulation of GO toxicity

**Figure 6:**
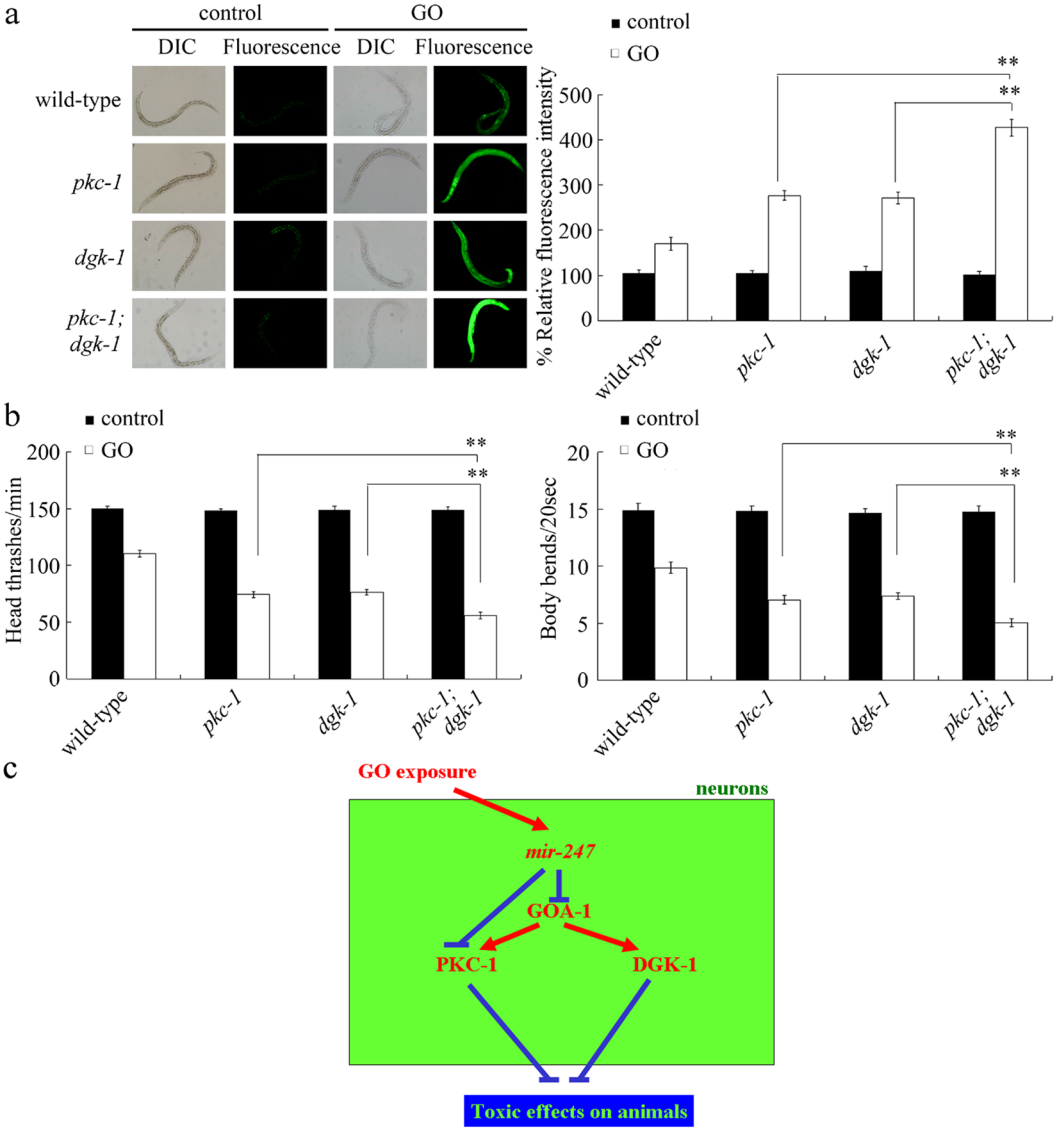
Genetic interaction of PKC-1 and DGK-1 in the regulation of GO toxicity. (**a**) Genetic interaction of PKC-1 and DGK-1 in the regulation of GO toxicity in inducing ROS production. GO exposure concentration was 10 mg/L. Prolonged exposure was performed from L1-larvae to adult day-1. Bars represent means ± SD. ^**^P < 0.01. (**b**) Genetic interaction of PKC-1 and DGK-1 in the regulation of GO toxicity in decreasing locomotion behavior. GO exposure concentration was 10 mg/L. Prolonged exposure was performed from L1-larvae to adult day-1. Bars represent means ± SD. ^**^P < 0.01. (**c**) A diagram showing the molecular basis for neuronal Gαo signaling in the regulation of GO toxicity in nematodes. A neuronal signaling cascade of *mir-247-*GOA-1-DGK-1/PKC-1 was raised to explain the molecular mechanism for GO toxicity induction in nematodes.

We further investigated the genetic interaction between the PKC-1 and DGK-1. After GO exposure, we observed the more severe GO toxicity in double mutant of *pkc-1(ok563);dgk-1(sy428)* compared with that in single mutant of *pkc-1(ok563)* or *dgk-1(sy428)* (Fig. 6a,b).

## Discussion

In this study, we first provide several lines of evidence to indicate the potential role of GOA-1 and PKC-1 as the targets for neuronal *mir-247* in the regulation of GO toxicity. First of all, expressions of both GOA-1 and PKC-1 could be suppressed by GO exposure, and their expressions could be further decreased by overexpression of neuronal *mir-247* in GO exposed nematodes (Fig. S1). Secondly, in nematodes, the phenotypes in GO exposed *goa-1(sa734)* or *pkc-1(ok563)* mutant were opposite to those in GO exposed *mir-247/797(n4505)* mutant (Fig. S2). Thirdly, we found that mutation of *goa-1* or *pkc-1* could effectively inhibit the resistance of *mir-247/797(n4505)* mutant to GO toxicity (Fig. 1). Moreover importantly, we observed that neuronal overexpression of *mir-247* did not influence the resistance of transgenic strain overexpressing neuronal *goa-1* lacking 3′-UTR or *pkc-1* lacking 3′-UTR to GO toxicity (Fig. 2), implying the binding of *mir-247* to the 3-UTR of *goa-1* or *pkc-1*. Previous study has identified the EGL-5 as the target for *mir-247* in the control of male tail development^46^. In this study, we identified the GOA-1 and the PKC-1 as the potential targets for *mir-247* during the control of GO toxicity formation in hermaphrodite nematodes.

GOA-1 activity is required for the regulation of asymmetric cell division in the early embryo, innate immunity, olfactory-mediated behaviors, and decision-making^42,43,47,48^. In this study, we further found the novel function of Gαo signaling in the control of nanotoxicity. In *C*. *elegans*, *goa-1* mutation induced a susceptibility of nematodes to GO toxicity (Fig. S2), implying that *goa-1-*encoded Gαo signaling negatively regulates GO toxicity.

The tissue-specific activity assays indicated that the neuronal GOA-1 regulates the GO toxicity (Fig. 3). In organisms, G protein coupled receptors (GPCRs), seven-transmembrane receptors, can sense the environmental signals or molecules outside the cell and activate the inside signal transduction pathways and, ultimately, the cellular responses by coupling with the G proteins^49^. The function of *goa-1-*encoded Gαo signaling in the neurons implies that certain GPCRs in the neurons may be activated or suppressed by GO exposure, and the affected neuronal GPCRs may further function through the *goa-1/*Gαo-mediated signaling cascade to regulate the GO toxicity.

In this study, GOA-1 could further act upstream of diacylglycerol kinase/DGK-1 and PKC-1 to regulate the GO toxicity. Under the condition of GO exposure, *goa-1* mutation decreased *dgk-1* and *pkc-1* expressions (Fig. 4a). Additionally, *dgk-1* or *pkc-1* mutation inhibited the resistance of transgenic strain overexpressing neuronal *goa-1* to GO toxicity (Fig. 5). *dgk-1* gene is expressed in most of the neurons. Therefore, a corresponding signaling cascade of GOA-1-DGK-1/PKC-1 can be raised to explain the molecular basis for neuronal *mir-247* in response to GO exposure (Fig. 6c).

Prolonged exposure to GO (≥10 μg/L) increased the *mir-247* expression^18^. Meanwhile, neuronal overexpression of *mir-247* induced a susceptibility to GO toxicity^18^. Therefore, the raised neuronal signaling cascade of *mir-247-*GOA-1-DGK-1/PKC-1 provides an important molecular mechanism for the potential GO toxicity induction in nematodes.

In this study, we further found that DGK-1 and PKC-1 functioned synergistically to regulate GO toxicity (Fig. 6a,b). PKC-1 plays a role in regulating function of nervous system, such as the neurotransmission^50^. This observation implies the possibility that, besides the normally considered downstream diacylglycerol kinase/DGK-1 signaling, the neuronal GOA-1/Gαo signaling may also regulate the GO toxicity by influencing the neurotransmission process. Our previous study has identified the NLG-1-PKC-1 signaling cascade in the regulation of GO toxicity^39^. Our results suggest that PKC-1 may act as an important link between the Gαo/GOA-1 signaling and the NLG-1 signaling in the regulation of GO toxicity. Additionally, PKC-1 may further act as the direct target for *mir-247* in the regulation of GO toxicity (Fig. 2). These results imply the potential crucial role of neurotransmission process in the toxicity induction in GO exposed nematodes.

In conclusion, we found that Gαo/GOA-1 and PKC-1 functioned as targets for neuronal *mir-247* in the regulation of GO toxicity. GOA-1 further acted upstream of both diacylglycerol kinase/DGK-1 signaling and PKC-1 signaling to regulate the GO toxicity. During the control of GO toxicity, DGK-1 and PKC-1 acted in parallel signaling pathways. Our data provide the important molecular basis for neuronal Gαo signaling in response to GO. Additionally, our results imply that certain neuronal GPCRs may sense the GO exposure, and the affected neuronal GPCRs may further regulate the functions of *goa-1/*Gαo-mediated signaling cascade to regulate the GO toxicity.

## Methods

### Preparation and characterization of GO

GO was prepared from natural graphite powder using a modified Hummer’s method^51^. The analysis of atomic force microscopy (AFM, SPM-9600, Shimadzu, Japan) indicated that the thickness of GO was approximately 1.0 nm in topographic height, corresponding to the property of one layer^18^. Sizes of most of the GO in K-medium after sonication (40 kHz, 100 W, 30 min) were in the range of 40–50 nm^18^. Assay on the Raman spectroscopy using a 632 nm wavelength excitation (Renishaw Invia Plus laser Raman spectrometer, Renishaw, UK) demonstrated the existence of G band at 1592 cm^−1^ and D band at 1326 cm^−1^, respectively^18^. The zeta potential of GO (10 mg/L) in the K-medium was −22.3 ± 2.7 mV^18^.

### *C*. *elegans* strains and culture

Nematodes used were wild-type N2, mutants of mir-247(n4505), goa-1(sa734), ceh-18(ok1082), pkc-1(ok563), dgk-1(sy428), and pkc-1(ok563);dgk-1(sy428), and transgenic strains of Ex(Punc-14-mir-247)^18^, goa-1(sa734)Ex(Punc-14-goa-1), goa-1(sa734)Ex(Pmyo-3-goa-1), goa-1(sa734)Ex(Pmyo-2-goa-1), Is(Punc-14-goa-1), Is(Punc-14-goa-1);dgk-1(sy428), and Is(Punc-14-goa-1);pkc-1(ok563). Some of the used strains were from Caenorhabditis Genetics Center. Nematodes were maintained on normal nematode growth medium (NGM) plates seeded with Escherichia coli OP50 at 20 °C^52^. The animals were lysed with a bleaching mixture (0.45 M NaOH, 2% HOCl) in order to separate the eggs from the adults, which allow us to obtain the age synchronous L1-larvae populations.

### Exposure and toxicity assessment

After sonication (40 kHz, 100 W, 30 min), GO was dispersed in the K medium to prepare a stock solution (1 mg/mL), which was diluted with the K medium to obtain the working solution. Prolonged exposure (from L1-larvae to adult day-1) was performed in liquid K medium at 20 °C in the presence of food (OP50).

Head thrash and body bend were used to assess the locomotion behavior. The method was performed under the dissecting microscope by eyes as described previously^6,53^. Fifty nematodes were examined per treatment.

Intestinal ROS production assay was performed as described previously^54^. After exposure, the nematodes were transferred to 1 μM 5′,6′-chloromethyl-2′,7′-dichlorodihydro-fluorescein diacetate (CM-H_2_DCFDA). After incubation (3 h in the dark), the nematodes were analyzed under a laser scanning confocal microscope (excitation wavelength, 488 nm; emission filter, 510 nm). Intestinal ROS signals were semi-quantified in comparison to autofluorescence. Sixty nematodes were examined per treatment.

### Reverse-transcription and quantitative real-time polymerase chain reaction (PCR)

Total RNA was isolated from the nematodes using Trizol reagent (Invitrogen, UK) according manufacturer’s protocol. Purity and concentration of RNA were evaluated by a ratio of OD260/280 using a spectrophotometer. The extracted RNA was used for the cDNA synthesis. After the cDNA synthesis, the relative expression levels of targeted genes were determined by real-time PCR in an ABI 7500 real-time PCR system with Evagreen (Biotium, USA). All reactions were performed in triplicate. Relative quantification of targeted gene was expressed as the ratio (targeted gene/reference gene *tba-1* encoding a tubulin). The related primer in formation is shown in Table S1.

### DNA constructs and germline transformation

To generate entry vector carrying promoter sequence, promoter region for *myo-2* gene specially expressed in pharynx, promoter region for *myo-3* gene specially expressed in muscle, or promoter region for *unc-14* gene specially expressed in neurons was amplified by PCR from wild-type *C*. *elegans* genomic DNA. The promoter fragment was inserted into pPD95_77 vector in the sense orientation. *goa-1/C26C6*.*2*.*1* cDNA containing or lacking 3′-UTR was amplified by PCR, and inserted into corresponding entry vector carrying the *myo-2*, *myo-3*, or *unc-14* promoter sequence. Transformation was performed by coinjecting testing DNA (10–40 μg/mL) and marker DNA of P*dop-1::rfp* (60 μg/mL) into the gonad of nematodes as described^55^. The related primer information for DNA constructs was shown in Table S2.

### RNA interference (RNAi)

RNAi assay was performed basically as described^54^. The nematodes were fed with *E*. *coli* strain HT115 (DE3) expressing double-stranded RNA for the examined gene. After grown in LB broth containing ampicillin (100 μg/mL), *E*. *coli* HT115 (DE3) expressing double-stranded RNA for the examined gene was plated onto NGM containing ampicillin (100 μg/mL) and isopropyl 1-thio-β-D-galactopyranoside (IPTG, 5 mM). L1 larvae were transferred onto certain RNAi plates until the nematodes became the gravid. The gravid adults were transferred to fresh RNAi-expressing bacterial lawns to let them lay eggs to obtain the second generation of RNAi population. The eggs were allowed to develop into L1-larvae for the toxicity assessment.

### Statistical analysis

Data in this article were expressed as means ± standard deviation (SD). Statistical analysis was performed using SPSS 12.0 software (SPSS Inc., Chicago, USA). Differences between groups were determined using analysis of variance (ANOVA), and probability levels of 0.05 and 0.01 were considered statistically significant.

## Supplementary information

Supporting Information

## References

[1] Ambros V, Lee RC, Lavanway A, Williams PT, Jewell D (2003). MicroRNAs and other tiny endogenous RNAs in *C*. *elegans*. Curr. Biol..

[2] Bartel DP (2004). MicroRNAs: genomics, biogenesis, mechanism, and function. Cell.

[3] Sokol NS (2012). Small temporal RNAs in animal development. Curr. Opin. Genet. Dev..

[4] Zhao L (2019). Dysregulation of *let-7* by PEG modified graphene oxide in nematodes with deficit in epidermal barrier. Ecotoxicol. Environ. Safety.

[5] Xiao G-S (2018). Biosafety assessment of water samples from Wanzhou watershed of Yangtze Three Gorges Reservoir in the quiet season in *Caenorhabditis elegans*. Sci. Rep..

[6] Wang, D.-Y. Nanotoxicology in *Caenorhabditis elegans*. Springer Nature Singapore Pte Ltd (2018).

[7] Yang R-L (2015). Insulin signaling regulates toxicity of traffic-related PM_2.5_ on intestinal development and function in nematode *Caenorhabditis elegans*. Toxicol. Res..

[8] Li Y-X (2011). Modulation of the assay system for the sensory integration of 2 sensory stimuli that inhibit each other in nematode *Caenorhabditis elegans*. Neurosci. Bull..

[9] Gonzalez-Moragas L (2017). Toxicogenomics of iron oxide nanoparticles in the nematode *C*. *elegans*. Nanotoxicology.

[10] Zhao L, Wan H-X, Liu Q-Z, Wang D-Y (2017). Multi-walled carbon nanotubes-induced alterations in microRNA *let-7* and its targets activate a protection mechanism by conferring a developmental timing control. Part. Fibre Toxicol..

[11] Yang R-L, Ren M-X, Rui Q, Wang D-YA (2016). *mir-231*-regulated protection mechanism against the toxicity of graphene oxide in nematode *Caenorhabditis elegans*. Sci. Rep..

[12] Khan AAP, Khan A, Asiri AM, Ashraf GM, Alhogbia BG (2017). Graphene oxide based metallic nanoparticles and their some biological and environmental application. Curr. Drug Metab..

[13] Georgakilas V (2016). Noncovalent functionalization of graphene and graphene oxide for energy materials, biosensing, catalytic, and biomedical applications. Chem. Rev..

[14] Li R (2018). Surface oxidation of graphene oxide determines membrane damage, lipid peroxidation, and cytotoxicity in macrophages in a pulmonary toxicity model. ACS Nano.

[15] Ema M, Hougaard KS, Kishimoto A, Honda K (2016). Reproductive and developmental toxicity of carbon-based nanomaterials: A literature review. Nanotoxicology.

[16] Yang K, Li Y, Tan X, Peng R, Liu Z (2013). Behavior and toxicity of graphene and its functionalized derivatives in biological systems. Small.

[17] Ibanez-Ventoso C, Vora M, Driscoll M (2008). Sequence relationships among *C*. *elegans*, *D*. *melanogaster* and human microRNAs highlight the extensive conservation of microRNAs in biology. PLoS ONE.

[18] Xiao G-S, Zhi L-T, Ding X-C, Rui Q, Wang D-Y (2017). Value of *mir-247* in warning graphene oxide toxicity in nematode *Caenorhabditis elegans*. RSC Adv..

[19] Friedman RC, Farh KK, Burge CB, Bartel DP (2009). Most mammalian mRNAs are conserved targets of microRNAs. Genome Res..

[20] Betel D, Koppal A, Agius P, Sander C, Leslie C (2010). Comprehensive modeling of microRNA targets predicts functional nonconserved and non-canonical sites. Genome Biol..

[21] Mizuno T (2004). The *Caenorhabditis elegans* MAPK phosphatase VHP-1 mediates a novel JNK-like signaling pathway in stress response. EMBO J..

[22] Feng H, Ren M, Chen L, Rubin CS (2007). Properties, regulation, and *in vivo* functions of a novel protein kinase D: *Caenorhabditis elegans* DKF-2 links diacylglycerol second messenger to the regulation of stress responses and life span. J. Biol. Chem..

[23] Jud MC (2008). Large P body-like RNPs form in *C*. *elegans* oocytes in response to arrested ovulation, heat shock, osmotic stress, and anoxia and are regulated by the major sperm protein pathway. Dev. Biol..

[24] Kang C, Avery L (2009). Systemic regulation of starvation response in *Caenorhabditis elegans*. Genes Dev..

[25] Esposito G, Amoroso MR, Bergamasco C, Di Schiavi E, Bazzicalupo P (2010). The G protein regulators EGL-10 and EAT-16, the Giα GOA-1 and the G(q)α EGL-30 modulate the response of the *C*. *elegans* ASH polymodal nociceptive sensory neurons to repellents. BMC Biol..

[26] Li H (2010). A proteomic view of *Caenorhabditis elegans* caused by short-term hypoxic stress. Proteome Sci..

[27] Château MT, Araiz C, Descamps S, Galas S (2010). Klotho interferes with a novel FGF-signalling pathway and insulin/IGF-like signalling to improve longevity and stress resistance in *Caenorhabditis elegans*. Aging.

[28] Yuan Y (2011). Dysregulated LRRK2 signaling in response to endoplasmic reticulum stress leads to dopaminergic neuron degeneration in *C*. *elegans*. PLoS One.

[29] Saifee O, Metz LB, Nonet ML, Crowder CM (2011). A gain-of-function mutation in adenylate cyclase confers isoflurane resistance in *Caenorhabditis elegans*. Anesthesiology.

[30] Takenaka M, Inoue H, Takeshima A, Kakura T, Hori T (2013). *C*. *elegans* Rassf homolog, *rasf-1*, is functionally associated with *rab-39* Rab GTPase in oxidative stress response. Genes Cells.

[31] Xie Y, Moussaif M, Choi S, Xu L, Sze JY (2013). RFX transcription factor DAF-19 regulates 5-HT and innate immune responses to pathogenic bacteria in *Caenorhabditis elegans*. PLoS Genet..

[32] De Vaux V (2013). The *Caenorhabditis elegans* LET-418/Mi2 plays a conserved role in lifespan regulation. Aging Cell.

[33] Tao L (2013). CAMKII and calcineurin regulate the lifespan of *Caenorhabditis elegans* through the FOXO transcription factor DAF-16. Elife.

[34] Goh GY (2014). The conserved Mediator subunit MDT-15 is required for oxidative stress responses in *Caenorhabditis elegans*. Aging Cell.

[35] Zhi L-T, Fu W, Wang X, Wang D-Y (2016). ACS-22, a protein homologous to mammalian fatty acid transport protein 4, is essential for the control of toxicity and translocation of multi-walled carbon nanotubes in *Caenorhabditis elegans*. RSC Adv..

[36] Zhi L-T, Ren M-X, Qu M, Zhang H-Y, Wang D-Y (2016). Wnt ligands differentially regulate toxicity and translocation of graphene oxide through different mechanisms in *Caenorhabditis elegans*. Sci. Rep..

[37] Tian Y (2016). Mitochondrial stress induces chromatin reorganization to promote longevity and UPR(mt). Cell.

[38] Xiao Y, Liu F, Zhao PJ, Zou CG, Zhang KQ (2017). PKA/KIN-1 mediates innate immune responses to bacterial pathogens in *Caenorhabditis elegans*. Innate Immun..

[39] Chen H, Li H-R, Wang D-Y (2017). Graphene oxide dysregulates Neuroligin/NLG-1-mediated molecular signaling in interneurons in *Caenorhabditis elegans*. Sci. Rep..

[40] Zhi L-T (2017). Graphene oxide induces canonical Wnt/β-catenin signaling-dependent toxicity in *Caenorhabditis elegans*. Carbon.

[41] Wu Q-L, Zhao Y-L, Zhao G, Wang D-Y (2014). microRNAs control of *in vivo* toxicity from graphene oxide in *Caenorhabditis elegans*. Nanomedicine: Nanotechnol. Biol. Med..

[42] Segalat LS, Elkes DA, Kaplan JM (1995). Modulation of serotonin-controlled behaviors by Go in *Caenorhabditis elegans*. Science.

[43] Mendel JE (1995). Participation of the protein Go in multiple aspects of behavior in *C*. *elegans*. Science.

[44] Land M, Islas-Trejo A, Freedman JH, Rubin CS (1994). Structure and expression of a novel, neuronal protein kinase C (PKC1B) from *Caenorhabditis elegans*. PKC1B is expressed selectively in neurons that receive, transmit, and process environmental signals. J. Biol. Chem..

[45] Bastiani, C. & Mendel, J. Heterotrimeric G proteins in *C*. *elegans*. *WormBook*, 10.1895/wormbook.1.75.1 (2006). 10.1895/wormbook.1.75.1 PMC4781550 18050432

[46] Zhang H, Emmons SW (2009). Regulation of the *Caenorhabditis elegans* posterior Hox gene *egl-5* by microRNA and the polycomb-like gene *sop-2*. Dev. Dyn..

[47] Los FCO, Ha C, Aroian RV (2013). Neuronal Goα and CAPS regulate behavioral and immune responses to bacterial pore-forming toxins. PLoS ONE.

[48] Anderson A, Laurenson-Schafer H, Partridge FA, Hodgkin J, McMullan R (2013). Serotonergic chemosensory neurons modify the *C*. *elegans* immune responses by regulating G-protein signaling in epithelial cells. PLoS Pathog..

[49] Trzaskowski B (2012). Action of molecular switches in GPCRs–theoretical and experimental studies. Curr. Med. Chem..

[50] Sieburth D, Madison JM, Kaplan JM (2007). PKC-1 regulates secretion of neuropeptides. Nat. Neurosci..

[51] Kovtyukhova NI (1999). Layer-by-layer assembly of ultrathin composite films from micron-sized graphite oxide sheets and polycations. Chem. Mater..

[52] Brenner S (1974). The genetics of *Caenorhabditis elegans*. Genetics.

[53] Wang D-Y (2014). Dopamine receptors antagonistically regulate behavioral choice between conflicting alternatives in *C*. *elegans*. PLoS ONE.

[54] Ding X-C (2018). Toxicity of graphene oxide in nematodes with deficit in epidermal barrier caused by RNA interference knockdown of *unc-52*. Environ. Sci. Technol. Lett..

[55] Mello C, Fire A (1995). DNA transformation. Methods Cell. Biol..

