# Supplementary material for: Dysregulation of Neuronal Gαo Signaling by Graphene Oxide in Nematode Caenorhabditis elegans
Source: Sci Rep. 2019 Apr 15;9:6026. doi: 10.1038/s41598-019-42603-1 (PMC6465305; doi:10.1038/s41598-019-42603-1)
Supplement: Supplementary file 1 — Supporting Information [file 41598_2019_42603_MOESM1_ESM.pdf]

Dysregulation of Neuronal Gαo Signaling by Graphene Oxide in Nematode *Caenorhabditis elegans*

Peidang Liu[a], Huimin Shao[a], Xuecheng Ding[a, b], Ruilong Yang[a, b], Qi Rui[b]& Dayong Wang[a, *]

[a]Medical School, Southeast University, Nanjing 210009, China

[b]College of Life Sciences, Nanjing Agricultural University, Nanjing 210095, China

[*]Correspondence and request for materials should be addressed to D.W. (email: dayongw@seu.edu.cn).

Supporting Information:

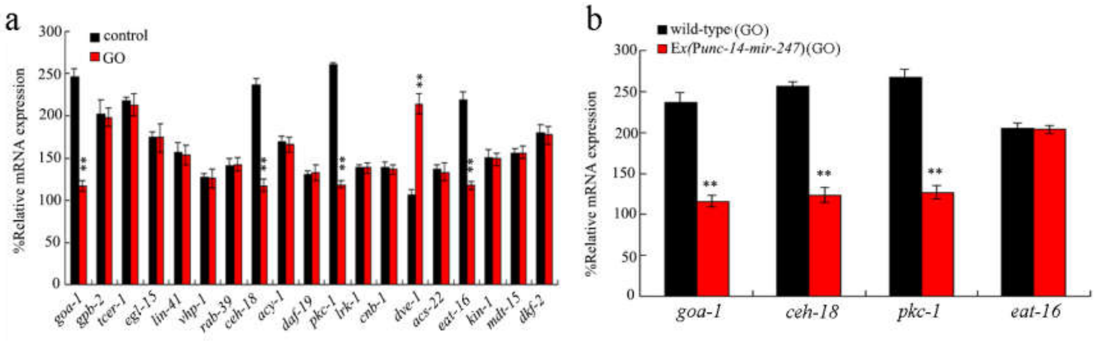

Figure S1. Screen of possible targets for neuronal *mir-247* in the regulation of GO toxicity. (**a**) Effect of GO exposure on gene expressions. (**b**) Effect of neuronal overexpression of *mir-247* on gene expressions in GO exposed nematodes. GO exposure concentration was 10 mg/L. Prolonged exposure was performed from L1-larvae to adult day-1. Bars represent means ± SD. $^{**}P < 0.01$ *vs* control or wild-type (GO).

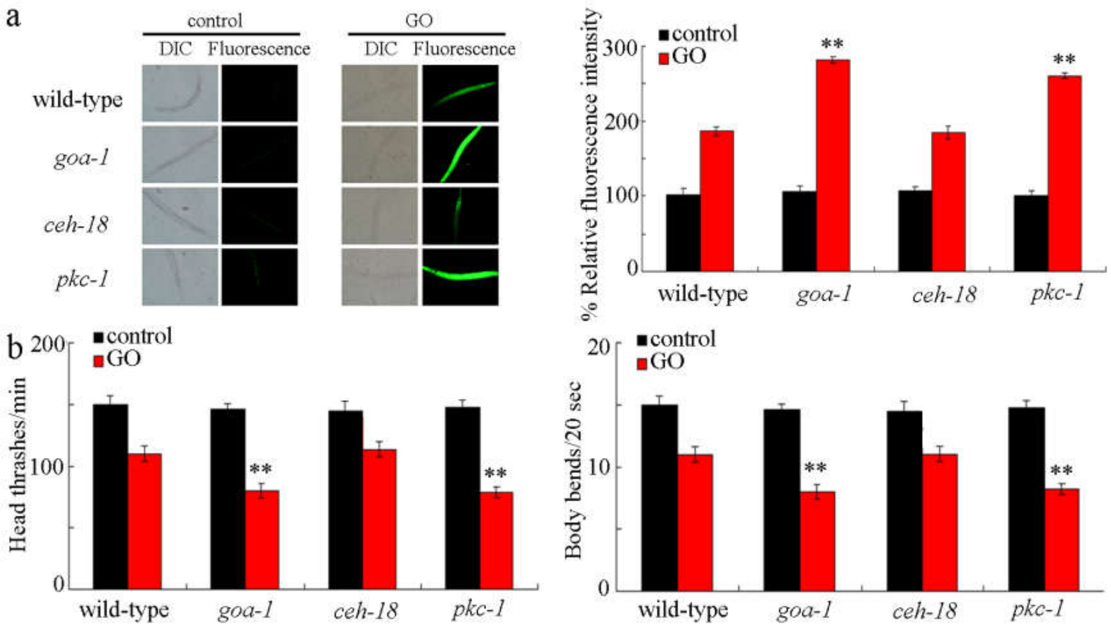

Figure S2. Effectof *goa-1*, *ceh-18*, or *pkc-1* mutation on GO toxicity in nematodes. (**a**) Effect of *goa-1*, *ceh-18*, or *pkc-1* mutation on GO toxicity in inducing intestinal ROS production. (**b**) Effect of *goa-1*, *ceh-18*, or *pkc-1* mutation on GO toxicity in decreasing locomotion behavior. GO exposure concentration was 10 mg/L. Prolonged exposure was performed from L1-larvae to adult day-1. Bars represent means ± SD. $^{**}P < 0.01$ *vs* wild-type.

Table S1 Primer information for qRT-PCR

Gene	Forward primer (5'-3')	Reverse primer(5'-3')
goa-1	ACCATGTCACAGGAAGAGCG	TCTGTGTCCTCCATTCGTGC
gpb-2	AAGCTCACCGGAATGGCTTT	TTGGTGGCGATGTACGAGAT
tcer-1	CGATGATTTTGGCGGCAGAG	ATCTTGTGGATTCGGCCCTG
eat-16	ATGCCACCGTTGACCAAGAT	GGAACCAGTTCAGCCTGTGA
lin-41	CACCACTTGGATCTCAGCCA	ACGCCAGACACATGTCCATC
vhp-1	CCCTTCGATCTCGCCAAACT	AGCTCCGCAACAGAAGTACC
rab-39	TGGGATACAGCAGGACAGGA	TAGGCACACGGATTGTGACC
ceh-18	GCGTTCAAAGTTGACCCCAC	GGAGGCCGATTCTCAGCTTT
acy-1	TCTTCCAATCGGTGTCGCAA	CATCCAGAAGCAGCTCCCAA
daf-19	TCGCTGCCAAGATGTGAACT	GACGGGGGTATGCTGTATGG
pkc-1	ACGCGACGCGAACAACTTTG	CGAACGACTGCAGGATCACTT
lrk-1	AACCCGAGACTCCGAACAAC	ATGCTCTTGCAACCATCGGA
cnb-1	TTCGATGAGGACGGAAACGG	ATCCAAAGTCCCGACCACAT
dve-1	CGTCGAAACTGTACTAGCAGC	CGTTTCCAGCGTATCAAGCC
acs-22	ATGCAGTGGTTTTGTACAAT	CTATAGCAAATGCACGTAAA
egl-15	TGCAGCTGCTGGAGAGAAAA	AATTCGGGTTTGCTCATGCT
kin-1	AAGTGCAAGTCAAGTTTTTCAAAG	CGTCCGATTCGGCGTAAATG
mdt-15	CCGTGTACGACAGCAGGAAA	TGGTCCACCATTCATCTGGC
dkf-2	GGCCACCGAAGAAGATTCCA	TTGCAACTTCTGCACGCATC
pkc-1	ACGCGACGCGAACAACTTTG	CGAACGACTGCAGGATCACTT
dgk-1	TCAGCCACAGTCTCGTAA	TTTCACCTCGTCTTTATCC
tba-1	TCAACACTGCCATCGCCGCC	TCCAAGCGAGACCAGGCTTCAG

Table S2. Primer information for DNA constructs.

Gene	Forward primer (5'-3')	Reverse primer(5'-3')
Punc-14	ACGAAGCTTTTCCCAACTGGCAATACT	ATACTGCAGCCACAAAAGTTGAGAGCA
Pmyo-2	CCCAAGCTTGGTGGTGGACAGTAACTG TCTGT	AGCTCTAGAATTTCTGTGTCTGACGATC GAGG
Pmyo-3	CTCAAGCTTCACTTCCGGCGCCCTGAAT CTAA	TAGGGATCCCATTTCTAGATGGATCTAG TGGTCGTGGG
goa-1/C26C6.2.1+ 3'-UTR	ATAGGATCCATGGGTTGTACCATGTCA	CGCGAATTCCAGTGACTAGGTGT
goa-1/C26C6.2.1- 3'-UTR	ATAGGTACCATGGGTTGTACCATGTCA	ATACTCGAGATACAAGCCGCATCCACG
pkc-1/F57F5.5a-3'-UTR	ATACCCGGGATGCTGTTCACAGGCACC	ATAGGTACCGTAGGTAAAATGCGGATT
